# #Dementia: An Evaluation of the Worldwide Dementia Twitter Network

**DOI:** 10.1093/geroni/igaa057.328

**Published:** 2020-12-16

**Authors:** Varun Ayyaswami, Divya Padmanabhan, Arpan Prabhu

**Affiliations:** 1 University of Maryland Baltimore, BALTIMORE, Maryland, United States; 2 University of New England College of Osteopathic Medicine, Biddeford, Maine, United States; 3 University of Arkansas for Medical Sciences, Little Rock, Arkansas, United States

## Abstract

Social media engages an international network of healthcare stakeholders. Our study characterized the #dementia community on Twitter, which we hypothesized has increased substantially in the last six years. Symplur Signals, a healthcare social media analytics platform, was used to analyze public #dementia tweets between 1/1/2014 and 12/31/2019. Spam or users unclassified by the platform were excluded. Tweet activity, content, user characteristics, engagement, associated hashtags, sentiment, and network analysis metrics were obtained. Exactly 2,149,494 tweets made by 105,938 users resulted in 21,460,413,770 impressions. There was a 44.6% and 29.1% compound annual growth rate of tweets and users, respectively. The five most frequently tweeted words were people, care, help, living, and support. Among users with identifiable locations, the United Kingdom, United States, and Canada had the greatest number of users. Healthcare associated individuals (17.8%), health advocacy organizations (17.6%), non-healthcare associated individuals (17.0%), and non-healthcare associated organizations (13.8%) were among the top 500 influencers based on tweet number. Hashtags related to caregiving (#Caregiving, #carers, #care, #caregivers, #caregiver, #dementiacare) and Alzheimer’s disease (#alzheimers, #Alzheimer, #Alz, #EndAlz) were among the top 15 associated hashtags. Analysis of the 1,000 most recent tweets shows more tweets with positive (71.0%) than negative (29.0%) sentiments. Network analysis mapping connections between users shows advocacy organizations (i.e. @ASAging) and other health individuals (i.e. @LEAD_Coalition) were central conversation hubs in 2019. The #dementia network discusses themes important to older adults. The network’s rapid growth has enabled increased dissemination of dementia-related information by both healthcare and non-healthcare associated individuals and organizations.

